# The indirect impact of COVID-19 pandemic on the utilization of the emergency medical services during the first pandemic wave: A system-wide study of Tuscany Region, Italy

**DOI:** 10.1371/journal.pone.0264806

**Published:** 2022-07-01

**Authors:** Vieri Lastrucci, Francesca Collini, Silvia Forni, Sara D’Arienzo, Valeria Di Fabrizio, Primo Buscemi, Chiara Lorini, Fabrizio Gemmi, Guglielmo Bonaccorsi

**Affiliations:** 1 Epidemiology Unit, Meyer Children’s University Hospital, Florence, Italy; 2 Department of Health Sciences, University of Florence, Florence, Italy; 3 Quality and Equity Unit, Regional Health Agency of Tuscany, Florence, Italy; 4 Medical Specialization School of Hygiene and Preventive Medicine, University of Florence, Florence, Italy; University of Turin, ITALY

## Abstract

**Background:**

Utilization of Emergency Medical Services (EMS) declined during COVID-19 pandemic, but most of the studies analyzed components of the EMS system individually. The study aimed to evaluate the indirect impact of COVID-19 pandemic on the utilization of all the components of the EMS system of Tuscany Region (Italy) during the first pandemic wave.

**Methods:**

Administrative data from the health care system of Tuscany were used. Changes in utilization for out-of-hospital emergency calls and emergency vehicle dispatched, emergency department (ED) visits, and patients being admitted from the ED to an inpatient hospital bed (hospitalizations from ED) during the first pandemic wave were analyzed in relation with corresponding periods of the previous two years. Percentage changes and 95%CI were calculated with Poisson models. Standardized Ratios were calculated to evaluate changes in in-hospital mortality and hospitalizations requiring ICU.

**Results:**

Significant declines were observed in the utilization of all the EMS considered starting from the week in which the first case of COVID-19 was diagnosed in Italy till the end of the first pandemic wave. During the epidemic peak, the maximum decreases were observed: -33% for the emergency calls, -45% for the dispatch of emergency vehicles, -71% for ED admissions. Furthermore, a decline of 37% for hospitalizations from ED was recorded. Significant decreases in ED admissions for life threatening medical conditions were observed: acute cerebrovascular disease (-36%, 95% CI: -43, -29), acute myocardial infarction (-42%, 95% CI: -52, -31) and renal failure (-42%, 95% CI: -52, -31). No significant differences were found between the observed and the expected in-hospital mortality and hospitalizations requiring ICU during the epidemic peak.

**Conclusion:**

All the components of the EMS showed large declines in their utilization during COVID-19 pandemic; furthermore, major reductions were observed for admissions for time-dependent and life-threatening conditions. Efforts should be made to ensure access to safe and high-quality emergency care during pandemic.

## Introduction

The SARS-CoV-2 virus was first reported in China and then rapidly spread across countries causing the global pandemic of the coronavirus disease 2019 (COVID-19). Countries world-wide were forced to implement large-scale measures to prevent disease transmission and to redefine the health-care provision system in order to strengthen the capacity to cope with a potential and unpredictable increase of COVID-19 cases. These measures have led to drastic changes in the pattern of health service utilization [1–3]. A large decrease in hospital utilization was observed during the first wave of COVID-19 pandemic, with the overall volume of hospitalizations steeply declining compared to previous years [3–6].

This fall of volume may be in part attributable to hospital efforts to reallocate the resources in order to be prepared to face a sudden surge of COVID-19, such as the cancellation of elective surgeries and other non-critical medical services [3, 7]. But puzzling declines in hospital and emergency department (ED) admissions for acute medical conditions were also observed, including decreases in presentations for acute myocardial infarction, stroke, pneumonia, and for exacerbation of chronic obstructive pulmonary disease and heart failure [8–14]. Although the management at home of less severe acute cases in the primary care setting or by remote care may have prevented some hospital or ED admissions, this shift in the provision of care can only partly account for the observed drop of cases as this setting cannot replace the treatment received during a hospital admission. Instead, the decrease in the ED or hospital utilization likely reflects the tendency for patients to defer care due to fear of contagion, even when they are acutely ill [3, 7, 15]. Indeed, several studies described a decline in ED admission driven by a fall in both emergent and non-emergent ED visits, suggesting that the decrease may also be due to patient reluctance to visit hospitals during the pandemic [16–18]. Thus, the fall in the utilization of emergency medical services (EMS) during the pandemic could portend substantial harm to public health and not simply the absence of need. This issue may be particularly true among disadvantaged communities, who have been severely hit by the pandemic itself and are dependent on EMS for a larger proportion of their care [7].

Two research studies in the US have shown a decrease in EMS volume during the pandemic but were limited regarding the EMS components studied and types of patient diagnoses [5, 6]. To date, evidence on how the COVID-19 pandemic has affected the utilization of EMS has been mainly limited to only one component of the broad spectrum of the EMS system of a region or a country, with most of the studies carried out on the in-hospital EMS (ED visits or hospital admissions) of a single hospital or a group of hospitals [11]. Such studies only provide a partial understanding of the secondary impact of the COVID-19 on the health-seeking behavior and health-service utilization of patients with acute medical conditions. Evidence from the complete set of out-of-hospital EMS, ED admissions, and hospitalizations from ED may help to better design the health care delivery system during the pandemic time and to identify groups of patients at risk for under-treatment of acute medical conditions.

The aim of the present study was to assess and characterize the indirect impact of COVID-19 pandemic on the utilization of the EMS system of the Tuscany Region (Italy) during the whole first pandemic wave (February—July 2020). In particular, variations in the pattern of utilization of all the components of the EMS system (out-of-hospital emergency calls, emergency vehicle dispatched, ED visits, and hospitalizations from ED) were analyzed in relation to the COVID-19 epidemic situation, with a specific focus on the socio-demographic characteristics and disease categories of patients. Furthermore, in order to understand changes in the illness severity of patients using the EMS during COVID-19 pandemic, the study analyzed whether there were variations in the outcomes of hospitalizations from the ED.

Italy was one of the first Western countries severely affected by the coronavirus pandemic. The first autochthonous Italian case was identified on 21 February 2020, while in Tuscany the first case of SARS-CoV-2 infection was detected on 24 February 2020. In Tuscany, the daily number of newly reported COVID-19 cases for the first pandemic wave reached its peak on April 3 (18.5 per 100 000 population) while the daily number of COVID-19 hospitalizations reached the highest value on March 21^st^ (151 new hospitalizations) [19].

The Italian government has dealt with the pandemic by planning a three-phased strategy to contain the circulation of SARS-CoV-2 [15]. The first phase, from March 11 to May 3 2020, coincided with the national lockdown. All non-essential services and activities—including schools—were suspended and all non-essential travel and contact with others were banned by the imposition of a “stay-at home” order. Furthermore, physical distancing rule and the obligation to wear a face mask when leaving home were introduced. The adopted measures were effective in containing COVID-19 epidemic. The second phase, from May 4 to June 3 2020, was characterized by the gradual reopening of services and business and by the easing of travel bans: free movement was granted to all citizens within their Region but movement across Regions was forbidden for non-essential reasons. In phase 3, physical distancing rule and face masks remained mandatory, schools remained closed until September 2020 but free movement within the whole national territory was restored and cinemas and theatres reopened [14, 20].

## Materials and methods

This study was conducted in accordance with the Helsinki Declaration. According to the Italian legislation (law 211/2003) and the regional procedures, the study does not need ethic approval as it is a purely observational study on routinely collected anonymous data.

The study had a cross-sectional design and was carried out on administrative data from the Tuscany Public Health Care System (THPCS). Tuscany is an administrative Region located in central Italy with an extension of about 23,000 square kilometers and a population of more than 3,7 million residents. The health care system in Italy is a regionally based national health service; the TPHCS provides universal health coverage for all the residents of Tuscany. TPHCS counts 34 general hospitals and 4 university teaching hospitals; in total TPHCS have 38 EDs [15, 21].

The following databases from the regional healthcare administrative data system were used for the study: enrolment registry, out-of-hospital EMS, ED registry, hospital discharge abstract, and death registry.

The primary outcome measures were the utilization of out-of-hospital EMS, ED visits, and hospitalizations from ED by non-COVID-19 patients. As for the out-of-hospital EMS, phone calls for emergency medical assistance to the emergency dispatch centers and the number of medical care units dispatched were considered. For all the outcome measures, data of COVID-19 patients were excluded from the analyses. The time frame considered for the study was from 1 January 2020 to the 28 June 2020 (considered as the end of the first pandemic wave in Tuscany); the outcomes were measured by week (Monday to Sunday) and by considering two distinct epidemic periods: the epidemic peak (from week 11 to week 14) and tail (from week 22 to week 25) periods.

ED admissions and hospitalizations from ED were characterized according to the following covariates: age, sex, nationality, comorbidities, mode of arrival at the emergency department (walk-in, ambulance), triage category (from code 1 –highest level of urgency—to 5—lowest level of urgency), principal cause of admission, urbanization level of residence (urban areas, suburban areas, rural areas, isolated rural areas, very isolated rural areas), type of emergency department (rural, basic, first level, second level, paediatric second level). Comorbidity was measured using the Charlson Comorbidity Index (CCI) [22]. Further details concerning the ED classification and the urbanization level of residence variables are reported in the supplementary materials (S1 Appendix). As far as the principal causes of admission are concerned, the top 25 medical conditions responsible for hospitalization through ED in 2018–2019 were considered. To group the admissions by medical condition, the Clinical Classifications Software (CCS) was used. The CCS was developed by the Agency for Healthcare Research and Quality for the Healthcare Cost and Utilization Project and is a diagnosis and procedure categorization scheme based on International Classification of Diseases, 9th Revision, Clinical Modification (ICD-9-CM) [23]. The CCS groups the ICD-9-CM multitude of codes into clinically meaningful and mutually exclusive disease categories. CCS has proven to be a good classification scheme for utilization studies [24–26]. The main diagnoses were used to attribute each admission into one of the CCS categories. The identified top 25 CCS categories represented 33% and 30% of all ED admissions occurred in 2020 and 2018–19, respectively; as for hospitalizations from ED, they represented 65% of all hospitalizations from ED both in 2020 and 2018–2019.

In order to understand changes in illness severity of patients using EMS during COVID-19 pandemic, the study analyzed in-hospital mortality, hospitalizations requiring ICU, and hospitalizations requiring surgery (defined as a hospitalization with DRG in the surgical partition) as secondary outcomes. If less seriously ill patients were disproportionately staying away from the hospital, we expected the rates of hospitalizations requiring intensive care unit (ICU) and in-hospital mortality to rise. Conversely, if seriously ill patients avoided seeking care as less seriously ill patients did, we expected to see no variations in the rate of hospitalizations requiring intensive care unit (ICU) and in-hospital mortality.

Percentage changes and their 95% confidence intervals (CIs) in the utilization of out-of-hospital EMS, ED visits, and hospitalizations from ED during the first pandemic wave were calculated (January—June 2020) in relation with the average utilization registered in the corresponding periods of the previous two years (2018–2019). Due to the implementation of a new triage classification system which occurred in 2019, it was not possible to perform year-to-year comparisons in ED admissions by triage category. For this reason, percentage changes and their 95% CIs in average weekly ED admissions by triage categories were calculated using the first six weeks of 2020 (weekly average ED admissions from week 1 to week 6) as reference period; this period was referred as pre-epidemic period of 2020 as it ended one week before the first Italian case of COVID-19 was reported.

Ninety-five percent confidence intervals (95% CI) and the statistical significance of the percentage changes were calculated using the Poisson model for all the considered periods. To compare in-hospital mortality, hospitalizations requiring ICU and hospitalizations requiring surgery between 2018–2019 and 2020, age, sex, and CCI standardized ratios (SR) were calculated. The indirect standardization was performed using the patients hospitalized from ED in 2018–19 as the standard population. For each analysis, an alpha level of 0.05 was considered significant. The statistical software Stata 14 SE (StataCorp LP, College Station, Texas) was used for the data analyses.

## Results

**Fig 1:**
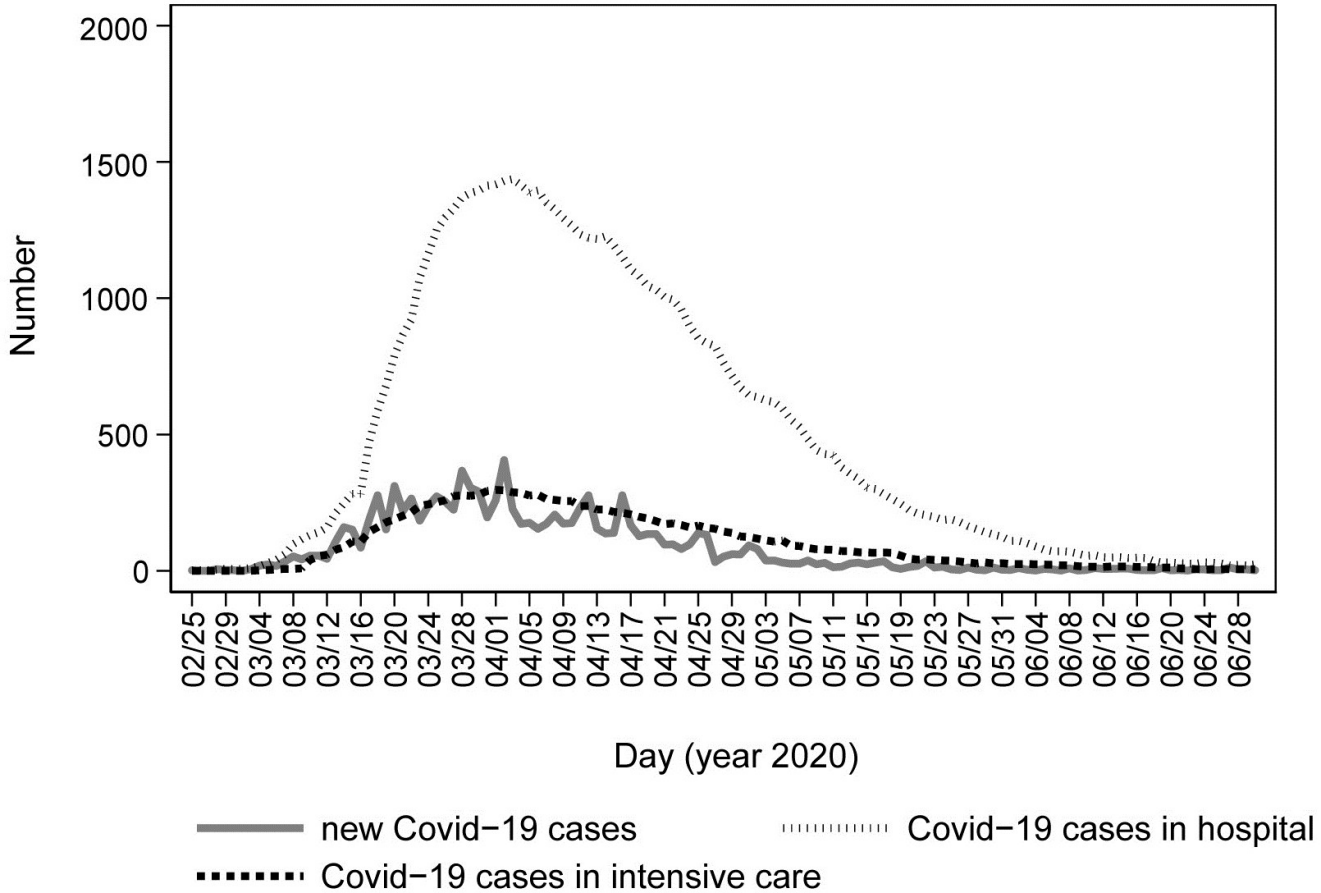
Number of COVID-19 cases registered in the general population of Tuscany Region during the first pandemic wave (January—June 2020).

Trend of COVID-19 pandemic observed in Tuscany region during the study period is reported in (Fig 1).

**Fig 2:**
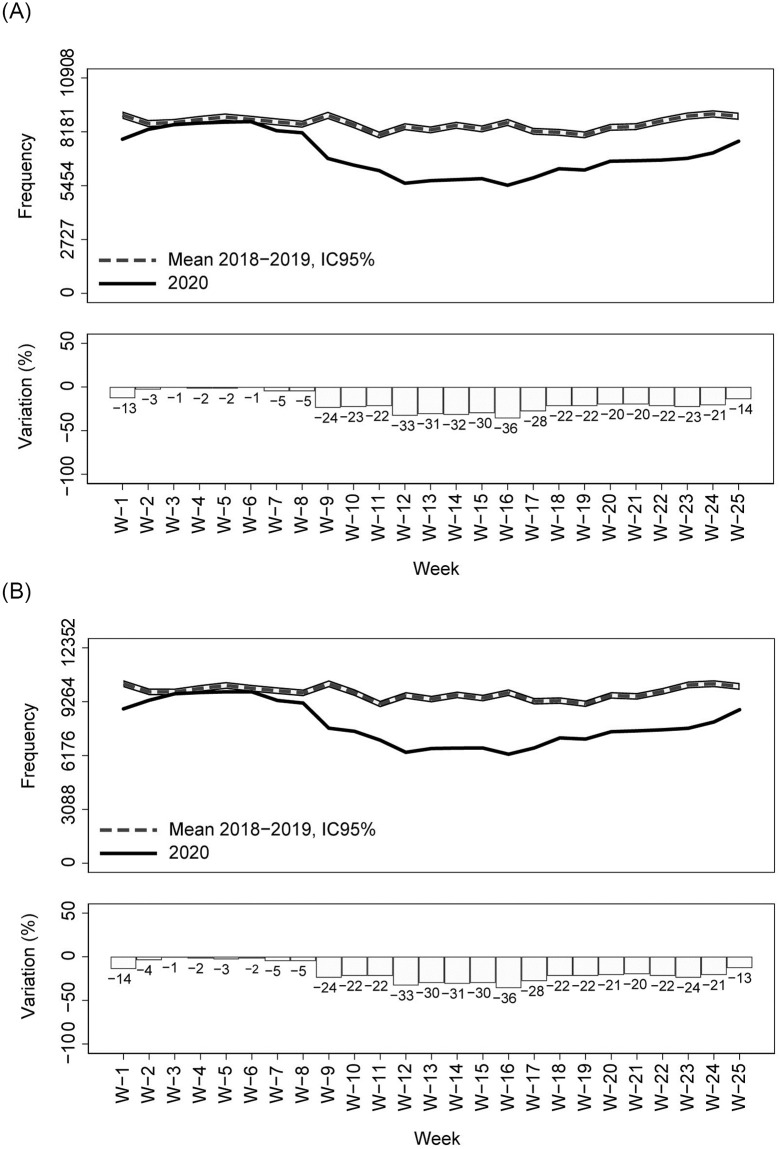
Utilization of out-of-hospital emergency medical services in Tuscany Region by week (year 2020 vs 2018–19); percentage changes and 95% CIs. (A) Weekly frequency of calls for emergency medical assistance; percentage changes and 95% CIs. (B) Weekly frequency of mobile medical care units dispatched; percentage changes and 95% CIs.

**Fig 3:**
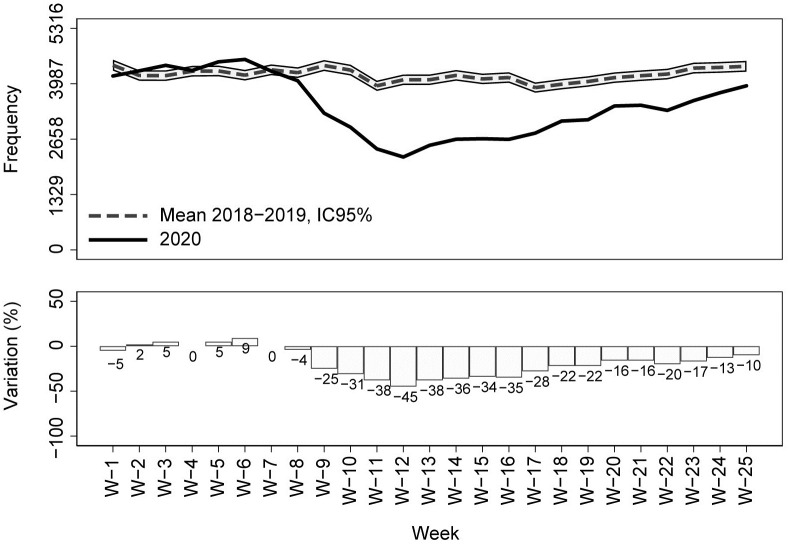
Emergency department admissions in Tuscany Region by week (year 2020 vs 2018–19); percentage changes and 95% CIs.

**Fig 4:**
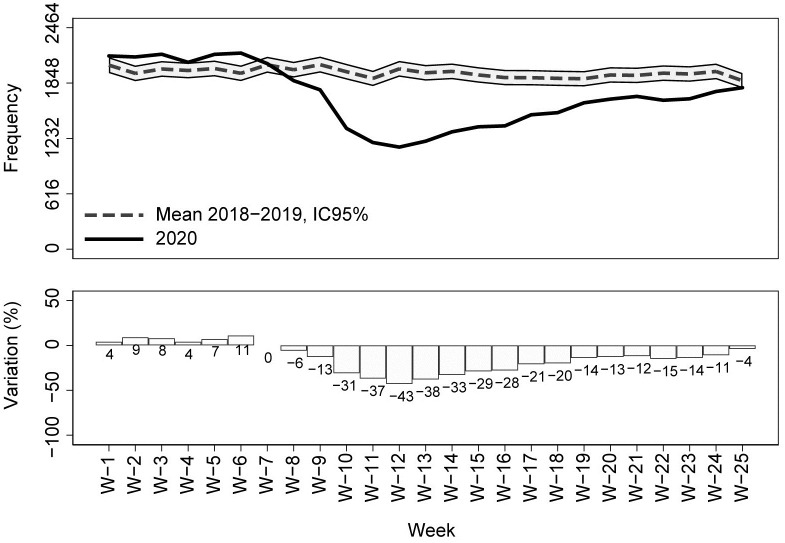
Hospitalizations from the emergency department in Tuscany Region by week (year 2020 vs 2018–19); percentage changes and 95% CIs.

Compared with the previous two years (2018–19) average, a drastic reduction in calls for emergency medical assistance and in the dispatch of mobile medical care units was observed starting from week 8 (the week in which the first COVID-19 case was reported in Tuscany Region) (Fig 2). Similarly, both the ED admissions and the hospital admissions from ED showed a steep decline compared to previous years starting from week 8 (Figs 3 and 4). During the COVID-19 peak period (week 11–14) (Fig 1), all the EMS services registered the maximum decreases in their utilization (-33% for the medical emergency calls, -45% for the dispatched emergency vehicles, -71% for ED admissions, and -37% for hospitalizations from ED in week 12) (Figs 2–4). After the COVID-19 peak period of the epidemic, the utilization of all the EMS gradually increased but remained significantly lower than the average value of the previous two years. During the last week of observation (week 25), the largest decrease was observed for ED admissions (-26% compared with 2018–2019), while medical emergency calls and the dispatch of medical emergency vehicles registered, respectively, significant percentage changes of -14% and -10% compared with 2018–2019. In the last week of observation, the number of hospitalizations from ED was not significantly different from those registered in the previous two years (-4%) (Figs 2–4).

**Fig 5:**
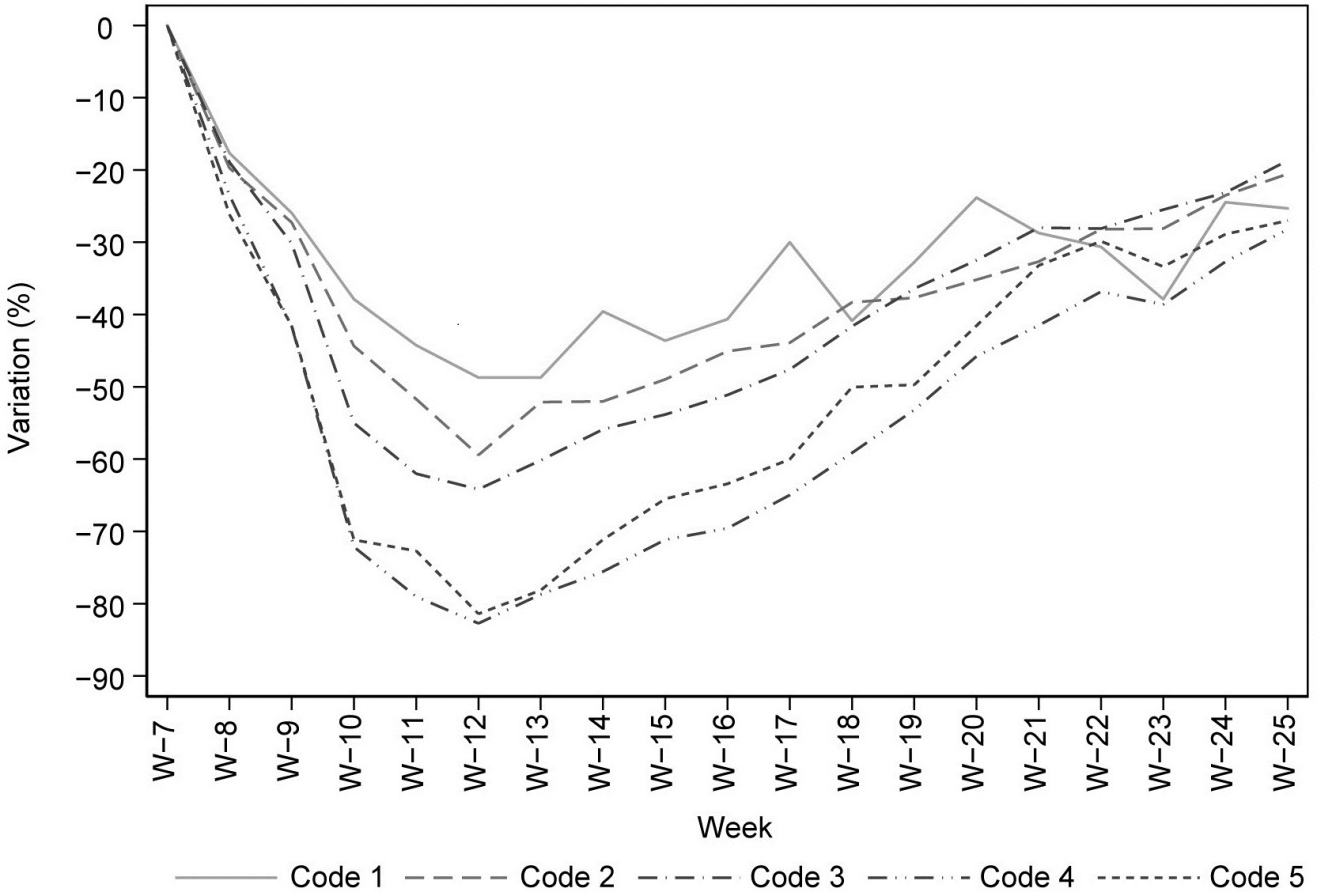
Percentage changes in emergency department admissions by triage category (Year 2020), reference period: Weekly average admissions registered in the pre-pandemic period (week 1 to 6 of 2020).

During the peak period of COVID-19 pandemic wave, a reduction of 73,041 ED admissions (-67%, 95%CI: -69%, -66%; lowest value: -71%, week 12) and of 3,017 hospitalizations from ED (-38%, 95%CI: -42%, -35%; lowest value: -43%, week 12) were observed (Tables 1 and 2). In the same period, significant decreases in ED admissions for all triage categories, including the highest priority codes (Code 1 and 2) were observed (Fig 5). The largest variation (about 80%, week 12) was observed for codes 4–5 (lowest priority), while the lowest reduction (over 40%, week 12) was observed for ED admissions with numeric code 1 (highest priority).

**Table 1:** Emergency department (ED) admissions in 2020 during the two considered epidemic periods (peak and tail) vs average ED admissions in 2018–19; percentage changes and 95% CIs (Poisson regression analysis).

	Epidemic peak				Epidemic tail			
	*2018–2019 (average)*	*2020*	*Percentage change (95%CI)*	*P*	*2018–2019 (average)*	*2020*	*Percentage change (95%CI)*	*P*
**Total admissions**	108,354	35,313	-67 (-69; -66)	<0.001	125,004	85,391	-32 (-34; -29)	<0.001
**Sex**								
Male	54,019	17,356	-68 (-70; -66)	<0.001	62,966	42,803	-32 (-35; -30)	<0.001
Female	54,335	17,957	-67 (-69; -66)	<0.001	62,038	42,588	-31 (-34; -29)	<0.001
**Age class**								
0–14 years	17,930	2,438	-86 (-88; -86)	<0.001	20,348	8,290	-59 (-62; -57)	<0.001
15–64 years	52,705	17,610	-67 (-69; -65)	<0.001	62,874	44,437	-29 (-32; -27)	<0.001
≥ 65 years	37,719	15,265	-60 (-62; -58)	<0.001	41,782	32,664	-22 (-26; -19)	<0.001
**Nationality**								
Italian	96,505	31,964	-67 (-69; -66)	<0.001	111,432	76,807	-31 (-34; -29)	<0.001
Foreign	11,849	3,349	-72 (-74; -71)	<0.001	13,572	8,584	-37 (-40; -35)	<0.001
**Mode of arrival at the ED**								
Ambulance	29,594	17,754	-40 (-42; -38)	<0.001	33,664	28,000	-17 (-20; -14)	<0.001
Walk-in	78,759	17,559	-78 (-79; -76)	<0.001	91,340	57,391	-37 (-40; -35)	<0.001
**Urbanization level of residence**								
Urban areas	49,982	15,922	-68 (-70; -67)	<0.001	57,865	39,051	-33 (-35; -31)	<0.001
Suburban areas	27,203	8,797	-68 (-70; -66)	<0.001	30,733	20,948	-32 (-35; -30)	<0.001
Rural areas	19,870	6,674	-66 (-69; -64)	<0.001	23,244	16,147	-31 (-34; -28)	<0.001
Isolated rural areas	8,950	3,084	-66 (-69; -63)	<0.001	10,430	7,255	-30 (-34; -28)	<0.001
Very isolated rural areas	2,350	836	-64 (-69; -61)	<0.001	2,734	1,990	-27 (-32; -23)	<0.001
**Type of emergency department**								
Second level	19,684	5,874	-70 (-73; -68)	<0.001	21,818.5	14,598	-33 (-36; -31)	<0.001
Paediatric second level	3,378	735	-78 (-81; -76)	<0.001	3,463.5	1,865	-46 (-50; -43)	<0.001
First level	56,731	18,279	-68 (-70; -67)	<0.001	66,167.5	45,528	-31 (-34; -29)	<0.001
Basic	24,322	9,266	-62 (-65; -60)	<0.001	28,314	19,980	-29 (-32; -27)	<0.001
Rural	4,240	1,159	-73 (-76; -70)	<0.001	5,241	3,420	-35 (-39; -31)	<0.001
**Charlson Comorbidity Index**								
0	84,535	24,579	-71 (-73; -69)	<0.001	99,356	66,405	-33 (35; -31)	<0.001
1	6,386	3,181	-50 (-53; -47)	<0.001	6,968	5,667	-19 (-22; -15)	<0.001
≥ 2	17,431	7,553	-57 (-59; -54)	<0.001	18,680	13,319	-29 (-32; -25)	<0.001

**Table 2:** Hospitalizations from the emergency department (ED) in 2020 during the two considered epidemic periods (peak and tail) vs average hospitalizations from ED in 2018–19; percentage changes and 95% CIs (Poisson regression analysis).

	Epidemic peak				Epidemic tail			
	*2018–2019 (average)*	*2020*	*Percentage change (95%CI)*	*P*	*2018–2019 (average)*	*2020*	*Percentage change (95%CI)*	*P*
**Total hospitalizations from ED**	7,848	4,831	-38 (-42; -35)	<0.001	8,317	7,450	-10 (-14; -6)	<0.001
**Sex**								
Male	3,548	2,085	-41 (-46; -38)	<0.001	3,807	3,321	-13 (-18; -8)	<0.001
Female	4,300	2,746	-36 (-40; -33)	<0.001	4,510	4,129	-8 (-14; -4)	= 0.001
**Age class**								
0–14 years	437	180	-59 (-65; -53)	<0.001	458	240	-48 (-54; -41)	<0.001
15–64 years	2,403	1,764	-27 (-32; -22)	<0.001	2,791	2,662	-5 (-11; 1)	= 0.126
≥ 65 years	5,008	2,887	-42 (-46; -39)	<0.001	5,068	4,548	-10 (-15; -6)	<0.001
**Nationality**								
Italian	7,352	4,465	-39 (-43; -37)	<0.001	7,746	6,930	-11 (-15; -7)	<0.001
Foreign	496	366	-26 (-37; -15)	<0.001	571	520	-9 (-18; 0)	= 0.065
**Mode of arrival at the emergency department**								
Ambulance	4.566	3,289	-28 (-31; -24)	<0.001	4,768	4,427	-7 (-11; -3)	= 0.002
Walk-in	3,282	1,542	-53 (-57; -48)	<0.001	3,549	3,023	-15 (-21; -9)	<0.001
**Urbanization level of residence**								
Urban areas	3,484	2,108	-39 (-44; -36)	<0.001	3,650	3,283	-10 (-16; -5)	= 0.001
Suburban areas	2,026	1,289	-36 (-41; -32)	<0.001	2,091	1,866	-11 (-17; -5)	= 0.001
Rural areas	1,524	928	-39 (-43; -35)	<0.001	1,685	1,544	-8 (-15; -2)	= 0.011
Isolated rural areas	659	420	-36 (-45; -28)	<0.001	718	622	-13 (-23; -3)	= 0.015
Very isolated rural areas	156	86	-45 (-54; -29)	<0.001	174	135	-22 (-33; -7)	= 0.005
**Type of emergency department**								
Second level	1,627	850	-48 (-52; -44)	<0.001	1,688	1,309	-22 (-28; -17)	<0.001
Paediatric second level	133	69	-48 (-55; -32)	<0.001	138	85	-38 (-45; -18)	<0.001
First level	4,174	2,564	-39 (-42; -35)	<0.001	4,450	4,195	-6 (-11; -1)	= 0.028
Basic	1,673	1,270	-24 (-30; -19)	<0.001	1,780	1,657	-7 (-13; -2)	= 0.019
Rural	242	78	-68 (-73; -53)	<0.001	262	204	-22 (-34; -9)	= 0.002
**Charlson Comorbidity Index**								
0	3,269	2,343	-28 (-33; -23)	<0.001	3,785	3,521	-7 (-12; -5)	<0.005
1	1,509	973	-36 (40; -31)	<0.001	1,561	1,441	-8 (-14; -1)	<0.023
≥ 2	3,070	1,515	-51 (-54; -47)	<0.001	2,971	2,488	-16 (-27; -10)	<0.001

When compared with the previous two years, the ED admissions and hospitalizations from ED during the epidemic peak significantly decreased (p<0.001) in each age group, nationality, Charlson Index category (0, 1, ≥2), type of ED, mode of arrival at the emergency department and neighborhood socio-economic status (Tables 1 and 2). The age group with the largest reduction was the class 0–14 years (admissions: -86%, 95% CI: -88, -86; hospitalizations from ED: -59%, 95% CI: -65, -53). The number of ED admissions and hospitalizations from ED with the walk-in arrival mode showed a larger reduction compared with the ambulance mode of arrival (Tables 1 and 2). ED admissions declined more in second level EDs (second level ED of paediatric hospital: -78%, 95% CI: -81, -76; second level ED: -70%, 95% CI: -73, -68) than in other types of ED, while the largest decline in hospitalizations was observed in rural ED (-68%, 95% CI: -73, -53) (Tables 1 and 2).

During the tail period of the epidemic, although the variations in ED admissions were of a lower extent compared with the peak period, they continued to be significantly decreased (p<0.001) in each category of all the variables considered (Table 1). In the same period, similar significant reductions were found for the hospitalizations from ED (Table 2). The only exceptions were age and nationality: no significant differences with the previous two years were observed in hospitalizations of patients with 15–64 years and of patients with a foreign nationality.

As for the principal cause of admission, during the epidemic peak significant decreases were found in ED admissions for all the medical conditions considered (p <0.001), except for normal pregnancy and/or delivery pregnancy and influenza-like illness (Table 3). ED admissions for influenza-like illness showed a significant increase (28%, 95% CI: 13–45, p <0.001). Among the admissions that showed a decrease, the admissions for pneumonia had the least variation (-24%, 95% CI: -32, -15). The greatest reductions were observed in admissions for abdominal pain (-76%, 95% CI: -78, -74), bronchitis (-68%; 95%CI: -74, -61), head trauma and syncope (respectively: -67%, 95% CI: -70, -65; -66%, 95% CI: -69, -61). Admissions for upper limb fracture and other external injuries decreased by 60% (95% CI: -65, -55) and 64% (95% CI: -68, -60), respectively. There were major reductions in accesses for acute cerebrovascular disease (-36%, 95% CI: -43, -29), myocardial infarction (-42%, 95% CI: -52, -31) and renal failure (-38%, 95% CI: -52, -21) (Table 3).

**Table 3:** Emergency department admissions by cause of admission (Clinical Classification Software category -CCS) in 2020 vs 2018–19 (average); percentage changes and 95% CIs (Poisson regression analysis).

*Clinical Classifications Software (CCS)*	Epidemic peak				Epidemic tail			
	*2018–2019 (average)*	*2020*	*Percentage change (95%CI)*	*P*	*2018–2019 (average)*	*2020*	*Percentage change (95%CI)*	*P*
131. Respiratory failure; insufficiency; arrest (adult)	1,339	682	-49 (-55; -43)	<0.001	1,161	723	-38 (-44; -31)	<0.001
108. Congestive heart failure; nonhypertensive	1,359	629	-54 (-58; -49)	<0.001	1,083	1,027	-5 (-14; 5)	= 0.927
122. Pneumonia	1,138	865	-24 (-32; -15)	<0.001	1,094	516	-53 (-58; -47)	<0.001
196. Normal pregnancy and/or delivery	1,331	1,276	-4 (-12; 4)	= 0.135	1,763	1,841	4 (-4; 14)	= 0.329
109. Acute cerebrovascular disease	851	543	-36 (-43; -29)	<0.001	848	799	-6 (-14; 3)	= 0.211
226. Fracture of neck of femur (hip)	588	425	-28 (-37; -17)	<0.001	578	584	1 (-10; 13)	= 0.872
246. Fever of unknown origin	1,489	905	-39 (-47; -31)	<0.001	2,125	811	-62 (-67; -57)	<0.001
100. Acute myocardial infarction	424	245	-42 (-52; -31)	<0.001	413	444	8 (-6; 22)	= 0.274
123. Influenza	660	846	28 (13; 45)	<0.001	534	275	-49 (-56; -40)	<0.001
244. Other injuries and conditions due to external causes	1,903	678	-64 (-68; -60)	<0.001	2,434	1,841	-24 (-29; -19)	<0.001
230. Fracture of lower limb	1,386	548	-60 (-65; -55)	<0.001	1,999	1,544	-23 (-29; -16)	<0.001
149. Biliary tract disease	685	342	-50(-57; -42)	<0.001	734	667	-9 (-18; 0)	= 0.057
251. Abdominal pain	4,529	1,075	-76 (-78; -74)	<0.001	4,683	3,052	-35 (-39; -31)	<0.001
59. Deficiency and other anemia	789	342	-57 (-64; -48)	<0.001	873	873	0 (-15; 17)	= 0.994
145. Intestinal obstruction without hernia	848	367	-57 (-60; -53)	<0.001	953	754	-21 (-29; -12)	<0.001
142. Appendicitis and other appendiceal conditions	279	127	-54 (-61; -48)	<0.001	312	229	-27 (-38; -13)	<0.001
229. Fracture of upper limb	2,739	974	-64 (-68; -60)	<0.001	3,485	2,787	-20 (-26; -14)	<0.001
130. Pleurisy; pneumothorax; pulmonary collapse	368	166	-55 (-64; -43)	<0.001	327	328	0 (-15; 19)	= 0.972
153. Gastrointestinal hemorrhage	500	249	-50 (-57; -42)	<0.001	484	399	-18 (-28; -6)	<0.001
125. Acute bronchitis	1,120	357	-68 (-74; -61)	<0.001	845	135	-84 (-87; -81)	<0.001
55. Fluid and electrolyte disorders	475	206	-57 (-64; -47)	<0.001	652	407	-38 (-45; -29)	<0.001
157. Acute and unspecified renal failure	269	166	-38 (-52; -21)	<0.001	329	318	-3 (-18; 14)	= 0.678
106. Cardiac dysrhythmias	2,198	814	-63 (-66; -60)	<0.001	1,997	1,774	-11 (-18; -4)	= 0.003
233. Intracranial injury	2,934	961	-67 (-70; -65)	<0.001	3,475	2,549	-27 (-30; -23)	<0.001
245. Syncope	1,821	627	-66 (-69; -61)	<0.001	2,050	1,375	-33 (-38; -27)	<0.001

During the tail period, the ED admission volume declined significantly for 16 of the 25 medical conditions considered (Table 3). In particular, the largest reductions were observed for acute bronchitis (-84%, 95% CI: -87, -81), for fever of unknown origin (-62%, 95% CI: -67, -57), for pneumonia and influenza-like illness (-53%, 95% CI -58, -47; -49%, 95% CI -56, -40, respectively). In the tail period, the admissions for acute cerebrovascular disease, myocardial infarction and renal failure were not significantly different from those of previous years (p> 0.05) (Table 3).

Table 4 reports the age, sex, and CCI standardized ratios for hospitalizations requiring ICU, hospitalizations requiring surgery, and in-hospital mortality. For these outcomes, no significant differences were found between the observed and the expected results during the COVID-19 epidemic peak period (SR 1.1, 95%CI 0.72–1.90 for in-hospital mortality; SR 1.0, 95%CI 0.83–1.23 for hospitalizations requiring ICU; and 0.9, 95% CI 0.85–1.12 for hospitalizations requiring surgery). During the epidemic tail, a significant reduction of in-hospital mortality was found (SR: 0.7, 95% CI: 0.51–0.95). Furthermore, a slight but significant increase in hospitalizations requiring surgery was observed (SR: 1.05, 95% CI: 1.03–1.25). In the same period, however, no significant variation was found in hospitalizations requiring ICU (SR 1.1, 95% CI: 0.86–1.49).

**Table 4:** Estimated standardized ratios (SR) for in-hospital mortality and for hospitalizations requiring ICU and surgery for the two considered epidemic periods (peak and tail).

	Epidemic peak				Epidemic tail			
	*Observed*	*Expected*	*SR*	*95%CI*	*Observed*	*Expected*	*SR*	*95%CI*
In-hospital mortality	373	354	1.1	0.72–1.90	382	522	0.7	0.51–0.95
Hospitalizations requiring ICU	489	469	1.0	0.83–1.23	751	691	1.1	0.86–1.49
Hospitalizations requiring surgery	1,204	1,276	0.9	0.85–1.12	2,018	1,944	1.05	1.03–1.25

## Discussion

Our study aimed to evaluate the secondary impact of large-scale containment measures for SARS-CoV-2 on the utilization of the EMS system of Tuscany Region (Italy) by non-COVID-19 patients during the first wave of COVID-19 (February–July 2020). To assess the impact on the utilization of EMS, the volumes of out-of-hospital EMS utilization, ED visits, and hospitalizations from ED were evaluated and compared with the average of the previous two years. In addition, hospitalizations requiring surgery, hospitalizations requiring ICU, and in-hospital mortality were assessed to evaluate variations in the severity of hospitalizations from the ED during the COVID-19 pandemic.

The utilization of out of hospital EMS, ED visits, and hospitalizations from ED by non-COVID-19 patients dramatically declined in March and April 2020 and then gradually rose back, but volumes of utilization remained significantly lower than the previous two years at the tail of the first pandemic wave. During the epidemic peak, ED admissions and hospitalizations from ED significantly decreased in all the patient groups considered. Significant decreases in ED admissions for all triage categories—including the highest priority codes—were observed. Furthermore, ED admissions for life threatening medical conditions such as acute cerebrovascular disease, acute myocardial infarction and renal failure were significantly lower during the epidemic peak. As for the severity of hospitalizations, no significant differences were found between the observed and the expected in-hospital mortality and hospitalizations requiring ICU during the epidemic peak, while a significant reduction of in-hospital mortality, but no significant variation in hospitalizations requiring ICU were observed in the tail of the epidemic.

Interestingly, the decline in the volume of EMS utilization started in the week in which the first case of COVID-19 was documented in Tuscany, with dramatic reductions observed afterwards during the national lockdown period. A possible explanation for this early impact is that the population probably was influenced by health risk messages from the media and national authorities rather than the actual epidemic situation; this tendency was already described in other studies [4, 7–14]. Several concurrent factors may have affected the utilization EMS during the pandemic. In particular, the fear of contracting SARS-CoV-2 in the hospital setting may have deterred patients from seeking hospital care. Furthermore, the increasingly stringent measures of containment and the sense of civic responsibility of the population may have played a relevant role in reducing the utilization of EMS especially in the context of an over-abundance of information—the so-called infodemic [27]—and conflicting messages from local and national authorities [7, 9]. Lastly, restriction measures played a direct role in reducing risk factors—such as road traffic accidents, falls and injuries and air-borne infectious diseases—for the incidence of several acute conditions usually treated in the EMS context; this is confirmed by the fact that injuries and fractures were among the causes of admissions that showed the largest decline in our study [8, 28–32].

Considering the different components of the emergency medical system, findings showed that they were hit differently by the pandemic during the general lock-down period. More specifically, ED visits had the largest reductions in utilization, when compared with out-of-hospital EMS. This phenomenon probably reflects the patient’s willingness to be cared for at home, especially during the time when SARS-CoV-2 was largely a nosocomial infection [2]. Furthermore, this larger decrease in in-hospital EMS utilization probably reflects also a significant reduction of inappropriate admissions as non-urgent triage categories were those that showed the highest volume reductions. Inappropriate ED admissions and hospitalizations are a well-known phenomenon and their large reduction has to be expected [33].

It should be pointed out that the very large entity of the reduction observed for ED admission with less severe triage codes and the concurrent and relevant decrease of the admissions for the highest priority of need codes probably indicates that also acute and critical patients avoided to seek care during the pandemic [3, 8–9, 12]. This is confirmed by the generalized reduction observed in the hospitalizations from ED that encompassed all the different patient subgroups considered. This suggests that during the first pandemic wave the population was not able to identify the need for urgent care and that the health system was unprepared to provide adequate responses for acute non-COVID-19 patients, thus it highlights several shortfalls that should be addressed for future pandemic. To achieve effective pandemic control measures and avoid their potential side effects a broad understanding and support from the population is essential [34]. In particular, clear and well-structured communication campaigns, strengthened and more integrated primary care support and the implementation of adaptive responses (e.g., teleconsultation, defined referral pathways) may ensure a higher public awareness and a better ability of healthcare system to intercept acute health needs of the population.

Observing the utilization of EMS by causes of admission, it is interesting to highlight two distinct tendencies. First, during the epidemic peak period major reductions occurred in admissions for time-dependent and life-threatening diseases, such as acute cerebrovascular disease, myocardial infarction, and acute renal failure; this might indicate a relevant increase in out-of-hospital mortality for such conditions. Though we are unable to quantify out-of-hospital mortality with the data available to us, other studies reported delayed care, worse health outcomes and increased mortality for time-dependent diseases during pandemic [10–13, 35, 36]. The second tendency that is worth to note is the increase of admissions for influenza-like illness during the epidemic peak period followed by the most significant reduction in the tail period. The most likely explanation of this is probably linked to the unpreparedness of the primary health-care during the first phases of the pandemic (e.g. lack of testing capacity, unavailability of PPE or adequate spaces for attending suspected COVID-19 cases). At that time, patients with influenza-like symptoms—driven by the fear of having COVID-19- may have visited the ED given the lack of responses provided by the primary health care services [37]. The above described tendencies have major implications for health service organization during pandemics; in particular, they highlight the need to organize distinct health care pathways that, on the one hand, allow to handle suspected COVID-19 patients at home or in the primary care context as much as possible and, on the other hand, allow the timely access to hospital care for patients with time-dependent and life-threatening diseases.

As far as the utilization of EMS services by patients’ subgroups is concerned, it is interesting to note that paediatric ED admissions and hospitalization were those most severely affected. This dramatic decline of EMS utilization in this age group is probably due to a combination of factors such as the reduction of trauma and injuries related to closure of schools, leisure and sport activities, and the reduction of recrudescence of diseases related to air pollution and of other infectious diseases [38–41]. Furthermore, it should be pointed out that inappropriate utilization of EMS is commonly reported for paediatric patients [38, 42], however delayed presentation for acute illnesses—probably linked to the reduced access to primary care—were reported during the first wave of the pandemic [38, 40].

Understanding the impact that such a dramatic change in the volumes and patterns of EMS utilization may have on the overall severity of cases hospitalized during the pandemic is complex, especially from routinely collected administrative data. However, the meaning of the unchanged rate of in-hospital mortality and hospitalizations requiring ICU during the peak of the epidemic, should not be underestimated, because it might be due to a situation where patients with serious and urgent conditions have delayed or avoided medical care as less seriously ill patients did. As a matter of fact, if patients affected by less serious conditions had disproportionately stayed away from the hospital, in-hospital mortality would have raised. Thus, the relative stability of indicators of hospitalization severity during the pandemic peak was probably accompanied by an increase of adverse outcomes and mortality at community level. Our findings confirm results of the study of Santi et al. in which the reduction of in-hospital mortality during the lockdown period was paired to a concurrent increase in out-of-hospital mortality [11]. Interestingly, during the tail of the epidemic the in-hospital mortality was lower compared with previous years, this is probably due to the return of less critical patients accumulated during the pandemic peak. The study presents several strengths and limitations. As for the strengths, data from the study describe the whole emergency care—the complete set of out-of-hospital and in-hospital EMS—provided in a wide and varied geographical area, in particular they can be considered representative of the whole EMS utilization of the population of Tuscany that counts of approximately 3.7 million inhabitants. Indeed, Tuscany emergency care is exclusively provided by the Regional public health care system. As far as the limitations are concerned, first, the cross-sectional design of the study does not allow to establish causal relationships. Secondly, the exhaustive interpretation of the study results is limited by the lack of data on mortality at community level and by the fact that the EMS utilization could have been influenced by a variety of factors that acted simultaneously during the pandemic (e.g. organizational, psychological, social and environmental). Lastly, due to a change in the triage coding system occurred during 2019, it was not possible to compare EMS utilization by triage codes for corresponding periods of different years. For this reason, admissions by triage codes were compared using the pre-epidemic period of the 2020 as a term of reference; however, it should be underlined that this analysis do not take into consideration the seasonality pattern related to the EMS utilizations.

In conclusion, out-of-hospital EMS, ED visits, and hospitalizations from ED showed large declines in their utilization during the first wave of COVID-19 pandemic; furthermore, major reductions were observed for admissions for time-dependent and life-threatening conditions. Efforts should be made by policy makers and public health practitioners to ensure access to safe and high-quality emergency care in a pandemic context.

## Supporting information

S1 Appendix Urbanization level of residence and emergency department classifications. (DOCX)
