# Supplementary material for: The indirect impact of COVID-19 pandemic on the utilization of the emergency medical services during the first pandemic wave: A system-wide study of Tuscany Region, Italy
Source: PLoS One. 2022 Jul 1;17(7):e0264806. doi: 10.1371/journal.pone.0264806 (PMC9249192; doi:10.1371/journal.pone.0264806)
Supplement: S1 Appendix — (DOCX) [file pone.0264806.s001.docx]

**S1 Appendix. Urbanization level of residence and emergency department classifications**

1. **Urbanization level of residence**

The classification of the urbanization level of residence adopted in this study was developed by the Department for the Economic Development and Cohesion of the Ministry of economic development of Italy; this classification is currently adopted for policy purposes and statistical reporting at national and regional level (2).

The municipality of residence were used to classify the urbanization level and the following category were adopted:

- Urban areas
- Suburban areas
- Rural areas
- Isolated rural areas
- Very isolated rural areas

Urban areas were defined as the municipalities that simultaneously offers:

- At least one high school for each of the specialization foreseen by Italian education system (lyceum; technical institute; professional institute)
- At least one hospital with a type 1 emergency department (i.e. italian health care system define a type 1 emergency department a 24-hour service with full resuscitation facilities provided of short-stay and cardiac intensive care units, shock room, multi-specialty emergency services and dedicated diagnostic imaging and laboratory services)
- At least one railway station of middle-small size with high volume of passengers (“Silver category” accordingly to the Italian railway network classification)

According to tertiles of the distribution of time necessary to reach the nearest urban area, the remaining municipalities were classified in peri-urban and rural and isolated rural areas. Areas above the 95^th^ percentile of the distribution of time necessary to reach the nearest urban area were defined as very isolated rural areas.

1. **Emergency department (ED) classification**

According to the Decree of the Italian Ministry of Health n.70 2/04/2015 (3), the ED in Italy are classified as follow:

1. **Rural emergency department**

ED of hospitals that are located in isolated and rural areas. Rural ED provides 24-hour emergency services and can count on the same hospital services of basic emergency departments (see below).

1. **Basic emergency department**

Catchment area between 80,000 and 150,000 inhabitants. Basic ED counts on the following hospital services: internal medicine, general surgery, orthopedics, anesthesiology and reanimation, intensive short-term observation. Basic ED counts on 24-hour availability of the following support services: radiology, laboratory, blood bank.

1. **First level emergency department**

Catchment area between 150,000 and 300,000 inhabitants.

First level ED counts on the following hospital services: internal medicine, general surgery, anesthesiology and reanimation, orthopedics and traumatology, obstetrics e gynecology, pediatrics, cardiology with cardiological intensive care unit, neurology, psychiatry, oncology, ophthalmology, otolaryngology, urology. First level ED counts on 24-hour availability of the following support services: radiology with at least one computerized axial tomography (CT) and ultrasound, laboratory, immunotransfusion service. For complex pathologies (such as trauma, cardiovascular, stroke), first level EDs have agreed protocol for consultation and patient transfer with second level ED. First level EDs are equipped with beds of intensive short-term and beds for sub-intensive therapy.

1. **Second level emergency department**

Catchment areas between 600,000 and 1,200,000 inhabitants.

Second level ED counts on the same hospital services as the first level ED as well as the following hospital services: neurosurgery, cardiac surgery, interventional hemodynamics, paediatric and neonatal intensive care, interventional radiology, thoracic surgery, maxillofacial surgery, plastic surgery, and other highly specialized disciplines.

**References**

1. <http://www.regione.toscana.it/-/censimento-popolazione-2011-dati-per-sezione-di-censimento-e-localita>
2. Barca F., Casavola P. and Lucatelli S. Strategia nazionale per le aree interne: definizione, obiettivi, strumenti e governance. Collana Materiali Uval, anno 2014, numero 31
3. Ministero della Salute. Decreto 2 aprile 2015, n. 70 Regolamento recante definizione degli standard qualitativi, strutturali, tecnologici e quantitativi relativi all'assistenza ospedaliera. (15G00084) (GU Serie Generale n.127 del 04-06-2015)
